# Developing a Validated Instrument to Measure Students’ Active Learning and Actual Use of Information and Communication Technologies for Learning in Saudi Arabia’s Higher Education

**DOI:** 10.3389/fpsyg.2022.915087

**Published:** 2022-06-16

**Authors:** Mohammed Abdullatif Almulla

**Affiliations:** Department of Curriculum and Instruction, Faculty of Education, King Faisal University, Al Ahsa, Saudi Arabia

**Keywords:** active learning and teaching methodologies, information and communication technologies (ICT), learning, higher education, structural equation modeling (SEM)

## Abstract

Higher education authorities have supplied information and communication technologies (ICTs) to guarantee that students use ICT to improve their learning and research outputs. ICT, on the other hand, has been proven to be underused, particularly by students. Therefore, we aimed to develop a new model to measure students’ active learning and actual use of ICT in higher education. To investigate this issue, the technology acceptance model and constructivism learning theory were verified and applied to evaluate university students’ use of ICT for active learning purposes. The participants in the study were 317 postgraduate and undergraduate students from four faculties at King Faisal University who consented to take part. The research data were analyzed using structural equation modeling (AMOS-SEM). Three specific components were used: the technology adoption model, constructivism learning, and active learning using ICT. The findings revealed that: (a) using ICTs for students’ interactivity, engagement, expected effort, subjective norm, and perceived ease of use has a direct positive impact on perceived enjoyment and usefulness; (b) perceived enjoyment and usefulness has a direct positive impact on active learning, attitude toward use, and behavioral intention to use ICTs; (c) active learning has a direct positive impact on attitude toward use, behavioral intention to use ICTs; and (d) active learning has a direct positive impact on attitude. Moreover, the results showed the mediator factors’ values positively “*R* square,” active learning (0.529), attitude toward use (0.572), behavioral intention to use (0.583), and actual ICT use (0.512) in higher education. Therefore, the results of the hypotheses developed a validated instrument to measure students’ active learning and actual use of ICTs in higher education in Saudi Arabia.

## Introduction

Information and communication technologies are information technologies and communication systems that use electronic equipment, particularly computers, to create, store, analyze, and transfer data. Teaching is regarded as one of the most difficult professions in contemporary culture, and ICT has taken root to provide a rich supply of knowledge. Through text, graphics, animation, music, and video, ICT provides a long-lasting influence on learning. Additionally, it fosters good social contact and improves learners’ interpersonal and intrapersonal abilities. The use of computer-based communication that includes classroom and e-learning teaching methods is referred to as ICT. Furthermore, ICT may achieve the goal by improving the quality, accessibility, and cost-effectiveness of the instruction delivery to students, providing learners with the advantages in dealing with globalization’s issues (John, 2015). Furthermore, ICT has a significant impact on a variety of professions, including medical, tourism, law, finance, business, and engineering. As a result, integration is not based on a single step; rather, it is based on a series of phases that provide comprehensive support for teaching–learning information resources. Since the 1960s, classroom response systems have been used in educational settings (Black and Wiliam, 1998). They began as voting devices on student seats at universities in the United States, in the form of fixed buttons and phone dial pads (Judson and Sawada, 2002).

Following that, the emergence of information technology, such as “clickers,” which are small remote control devices, raised the number of answers sent even more. The idea of employing digital technology to improve students’ learning skills in the future has obviously been embraced by academic study (Henderson et al., 2017; Pinto-Llorente et al., 2017). In addition to utilizing technology for learning, understanding how to use technology may be advantageous. According to the study, integrating tools and technology enables motivated learning and collaborative observations (Abdullah and Ward, 2016; Pinto-Llorente et al., 2017; Rezaei et al., 2018). The use of contemporary technology in higher education, particularly among undergraduate students, brings up new possibilities for critical thinking and collective review (Basogain et al., 2018). ICT is currently used by almost every business, organization, and institution. The use of ICT has revolutionized several aspects of human resources and computing methodologies (Gertrude, 2015). Higher education institutions have also acknowledged the need and have begun to focus on novel techniques to improve teaching and learning outcomes (Rasmussen and Hagen, 2015). Higher education institutions are working hard to incorporate cutting-edge teaching methods and equip campuses to meet the demands of contemporary trends and technology (Saliba, 2021). Universities prefer to plan for future issues to take full advantage of new and innovative technology prospects (Al Kurdi et al., 2020). To implement a technology educational environment, instructors must be actively involved (Tondeur et al., 2017). Only when the university professors are well-versed in and comprehend the latest technology solutions, they can use the correct instrument at the right time (Baturay et al., 2017).

Teachers’ expertise and criticism have been proven to be beneficial to students’ learning (Hina et al., 2020). University professors must have a positive attitude about what may be accomplished through technology-enabled learning and get a greater knowledge of the reality of students’ interactions with digital technology (Rashid and Asghar, 2016; Sumuer, 2021). It is well-known that not all learners effectively use ICTs in their learning; the reason for this is large investments in ICT integration in learning. Indicators of ICT in education are intended to improve educational results and student effectiveness (Asad et al., 2020; Butt et al., 2020). With the introduction of fast-changing technology, educators are not effectively using computers as they should be. It is important to remember that learners must know and master new skills as well as exercise new information (Jassim, 2020).

Most Saudi Arabian instructors and students are provided with computers and Internet access for personal use, but ICT integration in the classroom appears to be gradual. It moved quickly in the progressive countries. Due to limited resources, the adoption of ICT is the major phase in developing countries such as Saudi Arabia. Furthermore, teachers in rich nations improved their new skills, whereas students in underdeveloped countries improved their abilities faster than their teachers by using smartphones, iPads, and computers. It has been discovered that the major source of degradation in technology acquisition is not from students, but rather from professors. Not only are instructors hesitant to authorize technology, but other factors such as a lack of software and hardware, as well as teachers’ attitudes toward technology, provide a barrier. As a result, the goal of this research was to identify how ICT and online learning are integrated into Saudi Arabian higher education. In addition, the goal was to create a paradigm for ICT use.

## Research Model

**FIGURE 1:**
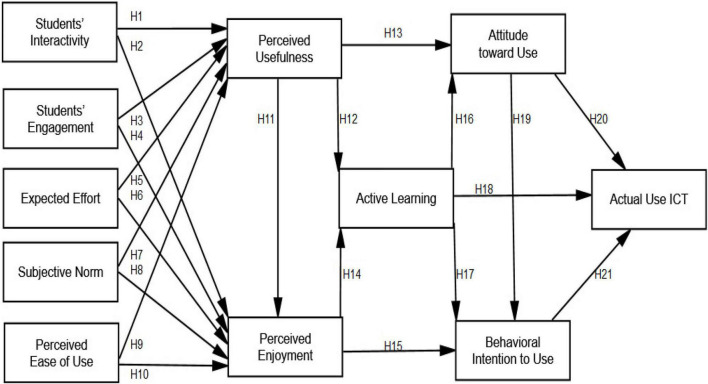
Research model and hypotheses.

The use of ICT in education enhances the teaching and learning process by assisting instructors and students and efficiently linking them to one another and a vast amount of information (Kreijns et al., 2013). There is a growing body of evidence supporting the use of ICT in education (Blackwell et al., 2014; Tondeur et al., 2017). The goal of this study is to come up with a list of characteristics that will boost the chances of effective ICT adoption in higher education. To accomplish so, researchers employed an enhanced version of Davis’ (1986, 1989) Technology Acceptance Model (TAM) to better comprehend the factors that influence ICT adoption in higher education. As a result, this research attempted to develop a new model by investigating the role of active learning and actual ICT use in higher education through students’ interactivity, engagement, expected effort, subjective norm, perceived ease of use, perceived enjoyment, perceived usefulness, attitude toward use, and behavioral intention to use ICTs (Figure 1).

### Students’ Interactivity

Although there is no general agreement on what constitutes interactive teaching, interactivity as a notion appears to have a significant role in reflecting what works in the classroom (Moyles et al., 2003). It is most frequently used in conjunction with whole-class instruction. Different types of interactivity can be classified using a learner influence scale over the course of an activity, ranging from a “lecture” style with no interaction between the teacher and the students to “funneling” questions, probing questions, uptake/focusing questions, and collective reflection, according to pedagogy and learning literature (Kennewell et al., 2008). Similarly, ICT has had a significant influence on the educational system, and it has been seen that ICT allows for more learning potential and a more dynamic learning environment in nations that have implemented ICT (Hennessy et al., 2010). According to several academics, ICT enhances the value of education by promoting good pedagogies that enable effective comprehension and promote involvement in learning. Many researchers, like Bingimlas (2009), agreed that ICT increases the student’s knowledge. ICT can also help with collaborative learning (Bindu, 2016), as well as the teaching–learning process, by enhancing interaction and knowledge reception. ICT has created a new environment that supports both individual and group learning and interactivity. Furthermore, effective and happy learning environments may be built using interactive online learning websites and applications (Goh and Sigala, 2020). The following hypotheses were suggested based on the discussion above:

H1: SI is positively associated with PU.

H2: SI is positively associated with PE.

### Students’ Engagement

The effort made by students to promote their psychological commitment to stay involved in the learning process to obtain knowledge and build critical thinking abilities is referred to as student engagement (Dixson, 2015). It is also tied to a student’s feeling of personal motivation in the course, which keeps them engaged, so they can connect with the course content, instructors, and classmates. In a nutshell, students’ engagement is essential for keeping students involved and supporting them in meeting their learning goals (Shea et al., 2006; Kehrwald, 2008). Richards (2011) emphasizes that meaningful learning occurs when students are actively participating, whereas Kuh (2003) defines student engagement as the amount of time and energy students spend on their educational activities. According to a study that used descriptive statistics, students’ interest and effective performance are also connected to regularity and tenacity in learning activities (Kennedy et al., 2015; Greller et al., 2017). The following hypotheses were suggested based on the discussion above:

H3: SE is positively associated with PU.

H4: SE is positively associated with PE.

### Effort Expectancy

The effort expectancy construct within each model is significant in both voluntary and mandatory usage contexts; however, each one is significant for the first time period before becoming non-significant over the extended and sustained usage (Venkatesh et al., 2003), which is consistent with previous research (Davis et al., 1989; Thompson et al., 1994; Agarwal and Prasad, 1998). To this end, we predict that the expectation of effort will be more visible in the early phases of each student’s behavioral desire to use ICT for learning. Increased ICT ease of use is expected to increase the usefulness of ICT as well as the behavioral intention to use it. Experienced users are undoubtedly less affected by the usefulness and ease of computer use. The expectation of effort was proposed as a direct predictor of behavior intention (Venkatesh et al., 2003). The assumption that effort expectation is a direct driver of behavioral intention to use ICT is supported by substantial evidence from recent research in the literature (Lwoga and Komba, 2015; Yakubu and Dasuki, 2019). In addition, numerous studies have found a substantial link between effort expectations and actual usage behavior (Jabeen et al., 2018; Moya et al., 2018). The following hypotheses were suggested based on the discussion above:

H5: EE is positively associated with PU.

H6: EE is positively associated with PE.

### Subjective Norm

The subjective norm is a person’s estimate of what others would think if they did or did not do something (Fishbein and Ajzen, 1977). The authors of a study (Venkatesh and Davis, 2000) found that subjective norm had a significant influence on the perceived usefulness and behavioral intentions toward required technology usage. When it comes to voluntary technology usage, subjective norm had a significant impact on perceived usefulness, but not on behavioral intentions. Based on the voluntary case results, it is expected that a similar conclusion will occur in this inquiry. Teo (2009) used the TAM to investigate technology acceptance among university students, and the results revealed that subjective norm significantly influenced perceived usefulness and perceived ease of use, but had no direct influence on intentions toward technology use, implying that subjective norm has an indirect influence on intentions toward technology use *via* perceived usefulness and perceived ease of use. The following hypotheses were suggested based on the discussion above:

H7: SN is positively associated with PU.

H8: SN is positively associated with PE.

### Perceived Ease of Use

The second TAM component, perceived ease of use, is described as an individual’s perception of how easy it would be to utilize a given technology (Davis, 1989). As a result, in this context, instructors’ perceived ease of use of computers is seen as a deciding element in their incorporation into the educational process. According to Watson (2006), the aptitude, abilities, and competencies of instructors in using computer technology for ICT-related tasks make its use considerably simpler. The majority of secondary school teachers, according to Chong et al. (2005), were focused on gaining the ICT skills needed to operate computers, with the authors suggesting that educators’ perceived ease of use directly led to the acceptability of technology in the teaching process. In addition, Askar et al. (2006) conducted a study of secondary school instructors in education to discover how innovative ICT-related activities are to them. Their findings demonstrated that instructors’ perceptions of the ease with which they might use ICT influenced the production of instructional materials in the classroom. Similarly, further empirical study has found that to successfully include ICT into knowledge distribution, educators must first see technology as simple to use (Wozney et al., 2006; Simonson, 2008; Andoh, 2012). The following hypotheses were suggested based on the discussion above:

H9: PEU is positively associated with PU.

H10: PEU is positively associated with PE.

### Perceived Usefulness

Perceived usefulness is the degree to which a user believes a system will improve his or her performance (Davis et al., 1989). Studies in the educational setting have backed up the applicability of the perceived usefulness concept. Watson (2006) discovered that knowing teachers’ perceptions of innovation is critical to successful technology adoption in the classroom. According to Bhattacherjee (2001) and Bennett and Bennett (2003), users would eventually use technology if they believe they will get expected benefits by doing so. The latter conducted a study on the effects of instructional technology on faculty members’ readiness to use technology in their teaching, which revealed that, rather than a lack of instructional facilities or education, the educator’s beliefs and reluctance to change were among the most important factors impeding ICT adoption. The following hypotheses were suggested based on the discussion above:

H11: PU is positively associated with PE.

H12: PU is positively associated with AL.

H13: PU is positively associated with AT.

### Perceived Enjoyment

Regardless of expected performance outcomes, judged enjoyment refers to how delightful an ICT-enabled activity is assessed to be (Van der Heijden, 2004). This construct may be thought of as a bi-perspective type of satisfaction that stems from using ICT with friends and helping others (Al-Rahmi et al., 2020a; Sayaf et al., 2021). Studying pleasure is a major indicator of intrinsic motivation as it shows how enjoyable and rewarding studying is for students (Krapp and Prenzel, 2011; Gong et al., 2020). Indeed, the control-value hypothesis suggests that students’ beliefs about their own skills, as well as whether learning is regarded as fun and worthwhile, impact learning enjoyment (Pekrun et al., 2007). Researchers discovered that negative emotional learning experiences were positively associated with avoidant coping strategies and negatively associated with academic performance, whereas positive emotional learning experiences were positively associated with problem-focused coping strategies and better performance (Krapp and Prenzel, 2011; Vierhaus et al., 2016). Students’ stated satisfaction is defined in this study as to how much they like using ICT for instructional reasons. The following hypotheses were suggested based on the discussion above:

H14: PE is positively associated with AL.

H15: PE is positively associated with BIU.

### Active Learning

Active learning has been pushed forward in higher education during the last decade, forcing lecturers to develop strategies to activate and promote student engagement (Tin, 2009). “The result of a teacher’s planned and conscious effort to urge pupils to participate explicitly in a lesson,” according to the definition of active learning (Pratton and Hales, 1986, p. 211). Rather than passively listening to lectures, it refers to strategies that actively engage students in the learning process (Blasco-Arcas et al., 2013; Alamri et al., 2020a). Higher knowledge acquisition, critical thinking, and material engagement are all possible with modern technology that supports active learning (Nicol et al., 2018). ICT-enabled collaboration between instructors and students encourages students to work together more dynamically and results in successful engagement with a topic’s material (Kay and LeSage, 2009). Incorporating ICT into active learning activities, such as asking students to reach a consensus in small groups, has also been demonstrated to assist students in achieving the benefits of active learning (Daniel and Tivener, 2016). According to Sun (2014), polling activities reduce graduate student anxiety, improve student performance, and retain students’ attention. Despite the fact that teachers may be hesitant to use ICT because of time constraints, technology has the potential to improve education (Farag et al., 2015). In order to achieve higher-order learning goals in classrooms, Hunsu et al. (2016, p. 114) believe that “emphasis must be placed on strategic lesson preparations as well as what happens in class while teaching.” The following hypotheses were suggested based on the discussion above:

H16: AL is positively associated with AT.

H17: AL is positively associated with BIU.

H18: AL is positively associated with AUI.

### Attitude Toward Use

According to the research, students’ attitudes about utilizing ICTs are influenced by their classroom (Fabunmi et al., 2007) or their commitment to and acceptance of their learning tasks (Thapa et al., 2021). According to Davis et al. (1989), perceived ease of use and the TAM impact perceived usefulness and, when combined, influence user attitudes to ICT usage. In a separate study, perceived value and ease of use were found as crucial markers for identifying virtual courses (Tan, 2019; Alamri et al., 2020b). The perceived ease of use influences students’ attitudes about using ICT as well as their behavioral intentions. In this study, students’ attitude toward ICT usage refers to how much they feel that using ICT improves their learning, which promotes their students’ attitude about using ICT. The following hypotheses were suggested based on the discussion above:

H19: AT is positively associated with BIU.

H20: AT is positively associated with AUI.

### Behavioral Intention to Use

Students’ positive views toward utilizing ICT tools had an impact on their behavioral intention to use them in this study. Studies have demonstrated that attitude is a powerful predictor of intention to use technology in a volitional scenario, when users have a choice whether or not to use technology (Teo et al., 2008; Teo, 2009). The idea of behavioral intention was born from the TRA’s Theory of Reasoned Action (TRA) (Fishbein and Ajzen, 1977). According to the construct’s definition, “a measure of the degree of one’s desire to engage in a given activity” (Ajzen, 1991). According to the study, a person’s behavioral intention has a direct impact on their actual technology usage (Ajzen, 1991). Both perceived ease of use and perceived usefulness were assumed to be influenced by external factors (Davis, 1989). TAM has been successfully employed by researchers to examine students’ behavioral intentions to use ICT for educational purposes throughout time (Shin and Kang, 2015; Sivo et al., 2018). According to a wide body of research, users’ intentions to utilize a system are largely impacted by their evaluations of its utility and ease of use (Al-Gahtani, 2016). The following hypothesis was suggested based on the discussion above:

H21: BIU is positively associated with AUI.

### Actual Use of Information and Communication Technologies

Many studies have attempted to stress and illustrate the beneficial functions of ICT use in student academic progress as ICT has advanced (Chiao and Chiu, 2018; Heinrich, 2021). Many studies have looked at the effects of ICT usage in learning on students’ academic success (Biagi and Loi, 2013; Al-Rahmi et al., 2020b), while others relied on empirical evidence from other surveys or experiments (Biagi and Loi, 2013; Rashid and Asghar, 2016; Al-Rahmi et al., 2020a; Heinrich, 2021). Regardless of the subjects under consideration, the majority of past research has concluded that there is a favorable association between ICT use and students’ academic success (Chiao and Chiu, 2018; Heinrich, 2021). The use of distant learning technology, according to Sayaf et al. (2021), has the potential to increase peer communication and cooperation, as well as coordinating ability and academic competence. The beneficial impact of ICT use on student academic success might be connected to the efficiency and productivity gained by using ICT in learning (Heinrich, 2021). Thanks to the availability of ICT, students may learn whenever and wherever they choose, at their own pace. Individuals may more easily get, exchange, and discuss a variety of learning resources and other extra information thanks to ICT. Researchers investigated not only the benefits and cons of adopting ICT in schools, but also the factors that aid or hinder real ICT use (Lee et al., 2005). The ICT abilities and capabilities of students will surely influence how they use technology in the classroom.

## Research Methodology

Questionnaires are the most common quantitative data gathering tool and are common in social science research. A five-point Likert scale questionnaire was used in this study as a quantitative data gathering instrument (Jamieson, 2004). Each question is a statement to which the responder must assign a number between 1 and 5 to indicate how strongly they agree or disagree with it (e.g., 1 = strongly agree, 2 = agree, 3 = neither agree nor disagree, 4 = disagree, and 5 = strongly disagree). Seven independent constructs were examined: students’ interactivity, students’ engagement, effort expectancy, subjective norm, perceived ease of use, perceived usefulness, and perceived enjoyment, as well as three dependent constructs: active learning, attitude toward use, and behavioral intention to use and AUI. Each of the constructs was measured through multiple items. Multiple items were used to assess each of the components.

### Sampling, Data Collection, and Data Analysis

The process of selecting a group of people from a population to represent the entire community in a research is known as sampling (McDonald et al., 2015). The most common way for getting solid and dependable results is probability sampling. Larger datasets increase the quality of study results by increasing generalizability and reliability (Collis and Hussey, 2013). This research emailed our survey to 370 students, and we received 285 answers. The students in the first year of an undergraduate degree at King Faisal University were picked at random. To maintain secrecy, the questionnaire was delivered online through Google Forms, and responders’ personal information was thereafter destroyed. The questionnaire components and associated items are listed in Table 3. The survey was done online, with SPSS 22.0 and Amos 23.0 used to analyze the findings. Structural equation modeling (SEM) was used to investigate the relationships between the key influencing components and active learning, attitude toward use, behavioral intention to use, and actual usage of ICT.

### Instrument Model

As stated in Table 3, a survey instrument was used to meet the study goals through an in-depth analysis. There were 11 constructs with 42 indicators. Students’ interactivity, students’ engagement, and active learning were proposed with the establishment of four items for each factor as recommended by Alyoussef et al. (2019) and Al-Rahmi et al. (2020a), the expected effort was proposed with the establishment of four items as recommended by Venkatesh et al. (2003), and the subjective norm was proposed with the establishment of four items as recommended by Venkatesh and Davis (2000). Also, perceived ease of use, perceived usefulness, and perceived enjoyment were proposed with the establishment of four items for each factor as recommended by Davis (1989). Attitude toward use and behavioral intention to use ICT were proposed with the establishment of three items for each factor as recommended by Davis (1989) and Teo (2009), and finally, the AUI was proposed with the establishment of four items as recommended by Alalwan et al. (2019) and Heinrich (2021).

## Data Analysis and Results

The demographic data are presented in Table 1. Among 317 useable questionnaires surveyed, 200 (63.1%) were from male respondents, while 177 were from female respondents (36.9%). Additionally, 211 (71.0%) were 17–22 years old, 48 (16.2%) were 23–27 years old, 10 (3.4%) were 28–30 years old, 11 (3.7%) were 31–34 years old, and 17 (5.7%) were more than 35 years old. Also, for the level of study, 256 (86.2%) were undergraduate students and 41 (13.8%) were postgraduate students. Finally, 98 (33.0%) faculties were from the Faculty of Education, 65 (21.9%) were from the Faculty of Art, 69 (23.2%) were from the Faculty of Law, and 65 (21.9%) were from the Faculty of Management, as given in Table 1.

**TABLE 1:** Demographic data.

	Factors	Frequency	Percent	Level of study	Factors	Frequency	Percent
Gender	Male	200	63.1		Undergraduate	256	86.2
	Female	117	36.9		Postgraduate	41	13.8
	Total	317	100.0		Total	317	100.0
Age	17–22	211	71.0	Faculty	Education	98	33.0
	23–27	48	16.2		Art	65	21.9
	28–30	10	3.4		Law	69	23.2
	31–34	11	3.7		Management	65	21.9
	>35	17	5.7		Total	317	100.0
	Total	317	100.0				

### Structured Equation Modeling

Pre-tests allow for the consideration of issues that cannot be expected during the administration of the questionnaire, assisting the researcher in obtaining better findings. Meanwhile, pilot testing tries to determine whether the research instrument will operate as a live project by implementing it with a small pilot population and identifying any flaws in the questions prior to a field launch. Initially, 40 questionnaires were distributed to the respondents, and the findings of the exploratory factor analysis revealed that each of the 11 factors was reliable and legitimate. A few small issues expressed during the pilot research were addressed, including the clarity of the instructions and questions, the overall design, and other minor observations. To ensure that the scales are meaningful, all ambiguities were eliminated. Factor loadings were used to establish construct validity, composite reliability, Cronbach’s alpha, and convergence validity for the model’s goodness of fit, as shown by Hair et al. (2012), as given in Table 2.

**TABLE 2:** The reliability coefficient for all variables.

Factors	Code	Pilot test	Final test
Students’ interactivity	SI	0.800	0.902
Students engagement	SE	0.792	0.892
Expected effort	EE	0.700	0.932
Subjective norm	SN	0.791	0.882
Perceived ease of use	PEU	0.712	0.900
Perceived enjoyment	PE	0.726	0.917
Perceived usefulness	PU	0.784	0.903
Active learning	AL	0.733	0.910
Attitude toward use	AT	0.797	0.907
Behavioral intention to use	BIU	0.735	0.923
Actual use of ICT	AUI	0.802	0.911

**TABLE 3:** Model fit evaluation.

Model fit	NFI	RFI	IFI	TLI	CFI	GFI	AGFI	RMR
Default model	0.928	0.920	0.948	0.942	0.948	0.932	0.926	0.031
Saturated model	1.000		1.000		1.000	1.000		0.000
Independence model	0.000	0.000	0.000	0.000	0.000	0.151	0.107	0.300

### Model Fit Measurement

**FIGURE 2:**
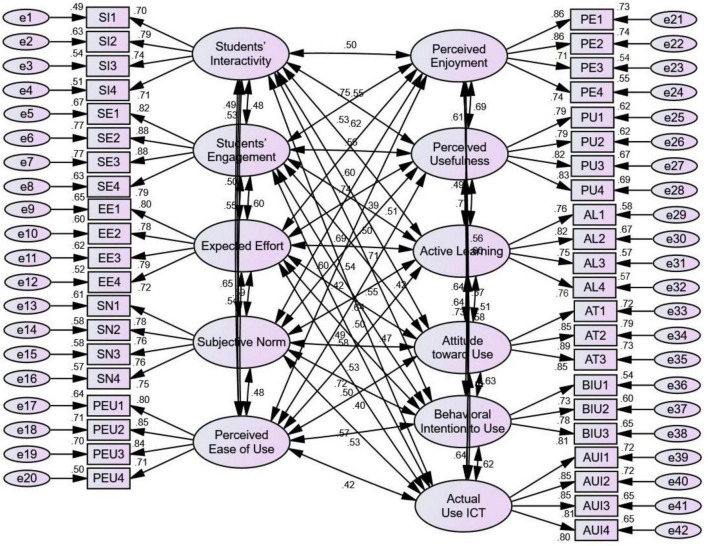
Measurement model.

The CMN/DF ratio in Table 3 is 2.287, which is lower than the necessary threshold (5.00). The RMR value below the threshold is 0.31 (0.05), AGFI (0.926) is a valid value, GFI (0.932) is a valid value, CFI (0.948) is a valid value, TLI (0.942) is a valid value, IFI (0.948) is a valid value, RFI (0.920) is a valid value, and NFI (0.928) is a valid value as suggested by Hair et al. (2012). Figure 2 shows all the items and factor values. This shows that the measurement model was acceptable and well-suited to the structural model (Table 3 and Figure 2).

### Reliability, Validity, and Measurement Model

The SEM-AMOS measurement model for each idea has its own set of characteristics, such as reliability and validity. Confirmatory factor analysis (CFA) and model fit were utilized to examine the intensity of the link direction using the structural model. Table 3 lists the factors of the measurement: The items of factor analysis meet the needed 0.700 level and above, the composite reliability (CR) of factor analysis meets the needed 0.800 level and above, the average variance extracted (AVE) of factor analysis meets the needed 0.500 level and above, and Cronbach’s alpha (CA) of factor analysis meets the needed 0.800 level and above. The results show all the items arranged from 0.889 to 0.699, the composite reliability arranged from 0.935 to 0.887, Cronbach’s alpha arranged from 0.932 to 0.882, and the average variance extracted arranged from 0.681 to 0.598, as given in Table 4.

**TABLE 4:** Reliability, validity, and measurement model.

No.	Items	Factors		Estimate	CR	CA	AVE	*R* square
1	SI1	←	Students’ interactivity	0.699	0.887	0.902	0.653	0.000
2	SI2	←		0.793				
3	SI3	←		0.737				
4	SI4	←		0.713				
5	SE1	←	Students’ engagement	0.817	0.905	0.892	0.603	0.000
6	SE2	←		0.880				
7	SE3	←		0.879				
8	SE4	←		0.792				
9	EE1	←	Expected effort	0.808	0.911	0.932	0.672	0.000
10	EE2	←		0.782				
11	EE3	←		0.787				
12	EE4	←		0.725				
13	SN1	←	Subjective norm	0.780	0.935	0.882	0.662	0.000
14	SN2	←		0.763				
15	SN3	←		0.760				
16	SN4	←		0.752				
17	PEU1	←	Perceived ease of use	0.800	0.909	0.900	0.611	0.000
18	PEU2	←		0.846				
19	PEU3	←		0.839				
20	PEU4	←		0.712				
21	PE1	←	Perceived enjoyment	0.857	0.923	0.917	0.681	0.502
22	PE2	←		0.863				
23	PE3	←		0.712				
24	PE4	←		0.738				
25	PU1	←	Perceived usefulness	0.781	0.889	0.903	0.598	0.498
26	PU2	←		0.790				
27	PU3	←		0.823				
28	PU4	←		0.832				
29	AL1	←	Active learning	0.763	0.919	0.910	0.607	0.529
30	AL2	←		0.818				
31	AL3	←		0.753				
32	AL4	←		0.764				
33	AT1	←	Attitude toward use	0.850	0.894	0.907	0.598	0.572
34	AT2	←		0.889				
35	AT3	←		0.851				
36	BIU1	←	Behavioral intention to use	0.732	0.910	0.923	0.625	0.583
37	BIU2	←		0.784				
38	BIU3	←		0.813				
39	AUI1	←	Actual use of ICT	0.852	0.907	0.911	0.672	0.512
40	AUI2	←		0.854				
41	AUI3	←		0.813				
42	AUI4	←		0.804				

### Measurement Validity Convergent

The distinctions between sets of ideas and their measurements are referred to as discriminant validity. The discriminant validity of all constructs was tested with values larger than 0.50 and significant at *p* = 0.001, as specified by Hair et al. (2012). The square root shared by objects in a single construct should be less than the similarities between items in the two constructions, and the findings were acceptable and arranged from 0.916 to 0.837, as shown in Table 5.

**TABLE 5:** Discriminant validity.

Factors	SI	SE	EE	SN	PEU	PE	PU	AL	AT	BIU	AUI
Students’ interactivity	0.898										
Students engagement	0.272	0.883									
Expected effort	0.239	0.328	0.837								
Subjective norm	0.254	0.335	0.343	0.916							
Perceived ease of use	0.260	0.282	0.369	0.297	0.855						
Perceived enjoyment	0.215	0.345	0.425	0.388	0.373	0.841					
Perceived usefulness	0.282	0.358	0.340	0.355	0.308	0.363	0.903				
Active learning	0.281	0.328	0.287	0.326	0.267	0.313	0.376	0.840			
Attitude toward use	0.243	0.259	0.288	0.284	0.292	0.348	0.293	0.287	0.853		
Behavioral intention to use	0.252	0.377	0.430	0.445	0.351	0.508	0.394	0.350	0.326	0.863	
Actual use of ICT	0.211	0.329	0.407	0.398	0.330	0.343	0.401	0.346	0.386	0.322	0.894

### Structural Model and Path Coefficient

**FIGURE 3:**
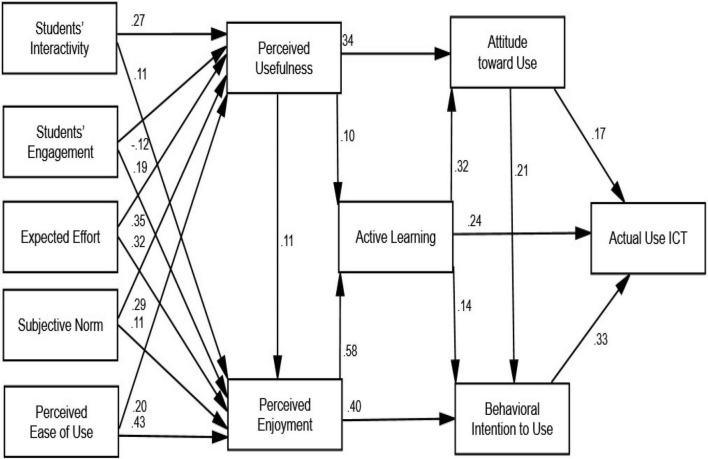
Path coefficient results.

Both the interaction and the effect of independent factors on the dependent variable are specified in the structural model (path coefficient). The maximum likelihood approach, in particular, may be used to extensively evaluate the complicated models and find numerous connections between multi-item elements, as well as the impact of moderating variables (Hair et al., 2012). The direct impact of the route coefficient on the latent predictor variable and expected variable is shown in Figure 3.

### Hypotheses Testing Results

**FIGURE 4:**
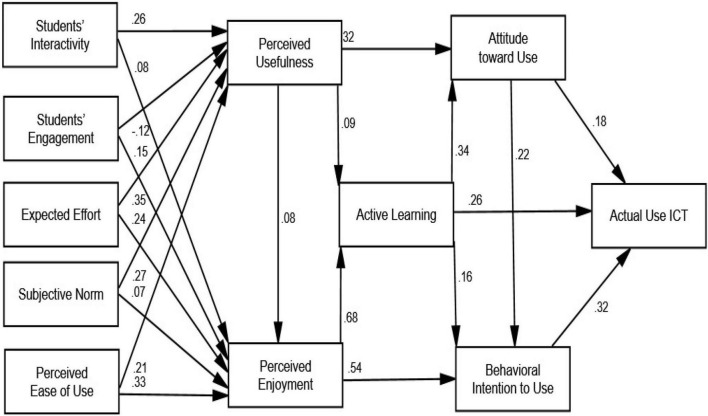
Path *T*-values results.

Based on the results shown in Table 5, the relationship between students’ interactivity and perceived usefulness (β = 0.259; C.R. = 8.534, *p* < 0.000) was accepted, and the relationship between students’ interactivity and perceived enjoyment (β = 0.083; C.R. = 3.758, *p* < 0.000) was accepted. Similarly, the relationship between students’ engagement and perceived usefulness (β = 0.124; C.R. = 4.249, *p* < 0.000) was accepted, and the relationship between students’ engagement and perceived enjoyment (β = 0.147; C.R. = 7.012, *p* < 0.000) was accepted. Also, the relationship between expected effort and perceived usefulness (β = 0.353; C.R. = 10.570, *p* < 0.000) was accepted and the relationship between expected effort and perceived enjoyment (β = 0.239; C.R. = 9.669, *p* < 0.000) was accepted. Moreover, the relationship between subjective norm and perceived usefulness (β = 0.268; C.R. = 9.194, *p* < 0.000) was accepted, and the relationship between subjective norm and perceived enjoyment (β = 0.075; C.R. = 3.491, *p* < 0.000) was accepted. Additionally, the relationship between perceived ease of use and perceived usefulness (β = 0.205; C.R. = 5.816, *p* < 0.000) was accepted, and the relationship between perceived ease of use and perceived enjoyment (β = 0.334; C.R. = 13.147, *p* < 0.000) was accepted. Furthermore, the relationship between perceived usefulness and perceived enjoyment (β = 0.082; C.R. = 4.164, *p* < 0.000) was accepted, the relationship between perceived usefulness and attitude toward the use of ICT (β = 0.325; C.R. = 15.036, *p* < 0.000) was accepted, and the relationship between perceived usefulness and active learning (β = 0.087; C.R. = 4.322, *p* < 0.000) was accepted. As well, the relationship between perceived enjoyment and active learning (β = 0.685; C.R. = 27.996, *p* < 0.000) was accepted and the relationship between perceived enjoyment and behavioral intention to use ICT (β = 0.536; C.R. = 15.925, *p* < 0.000) was accepted. Furthermore, the relationship between active learning and attitude toward the use of ICT (β = 0.344; C.R. = 13.593, *p* < 0.000) was accepted, the relationship between active learning and behavioral intention to use ICT (β = 0.156; C.R. = 4.912, *p* < 0.000) was accepted, and the relationship between active learning and AUI (β = 0.261; C.R. = 9.355, *p* < 0.000) was accepted. In addition, the relationship between attitude toward the use of ICT and behavioral intention to use ICT (β = 0.224; C.R. = 8.592, *p* < 0.000) was accepted, the relationship between attitude toward the use of ICT and AUI (β = 0.177; C.R. = 6.684, *p* < 0.000) was accepted, and the relationship between behavioral intention to use ICT and AUI (β = 0.319; C.R. = 12.570, *p* < 0.000) was accepted, as given in Figure 4 and Table 6.

**TABLE 6:** Hypotheses testing results.

No.	Relationships			Estimate (β)	S.E.	C.R.	*P*	Results
H1	PU	←	SI	0.259	0.030	8.534	0.000	Supported
H2	PE	←	SI	0.083	0.022	3.758	0.000	Supported
H3	PU	←	SE	0.124	0.029	4.249	0.000	Supported
H4	PE	←	SE	0.147	0.021	7.012	0.000	Supported
H5	PU	←	EE	0.353	0.033	10.570	0.000	Supported
H6	PE	←	EE	0.239	0.025	9.669	0.000	Supported
H7	PU	←	SN	0.268	0.029	9.194	0.000	Supported
H8	PE	←	SN	0.075	0.021	3.491	0.000	Supported
H9	PU	←	PEU	0.205	0.035	5.816	0.000	Supported
H10	PE	←	PEU	0.334	0.025	13.147	0.000	Supported
H11	PE	←	PU	0.082	0.020	4.164	0.000	Supported
H12	AT	←	PU	0.325	0.022	15.036	0.000	Supported
H13	AL	←	PU	0.087	0.020	4.322	0.000	Supported
H14	AL	←	PE	0.685	0.024	27.996	0.000	Supported
H15	BIU	←	PE	0.536	0.034	15.925	0.000	Supported
H16	AT	←	AL	0.344	0.025	13.593	0.000	Supported
H17	BIU	←	AL	0.156	0.032	4.912	0.000	Supported
H18	AUI	←	AL	0.261	0.028	9.355	0.000	Supported
H19	BIU	←	AT	0.224	0.026	8.592	0.000	Supported
H20	AUI	←	AT	0.177	0.027	6.684	0.000	Supported
H21	AUI	←	BIU	0.319	0.025	12.570	0.000	Supported

## Factors Described and Analyzed

**FIGURE 5:**
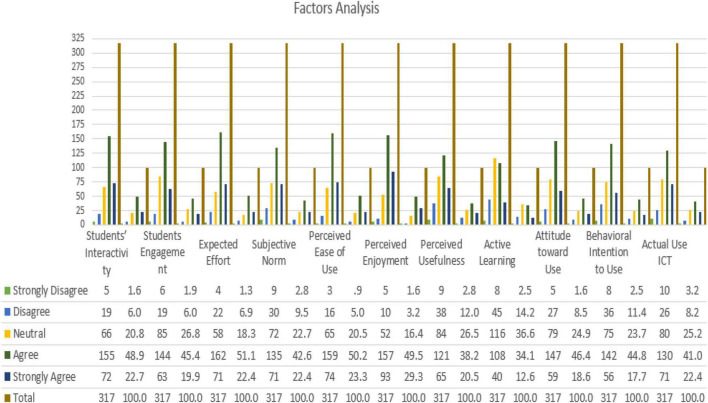
Factors described and analyzed.

The standard deviation (SD) and mean are the two statistics to describe the measurements in the research model. The majority of the data points are close to the mean when the standard deviation is low. The data are more distributed if the standard deviation is high. As a consequence, as shown in Figure 5, all values were accepted, and the majority either agreed or strongly agreed, meaning that the role of active learning and AUIs affected the education through students’ interactivity, students’ engagement, expected effort, subjective norm, perceived ease of use, perceived enjoyment, perceived usefulness, attitude toward use, and behavioral intention to use ICTs for education, as shown in Figure 5.

### Discussion and Implications

While there have been varied results about the role and impact of ICTs in learning, the results of most prior research have shown that ICT use for learning has a favorable influence. ICT use for learning, for example, has favorable and substantial benefits on students’ academic performance, according to Chiao and Chiu’s (2018) study. Sayaf et al. (2021) discovered a relationship between computer self-efficacy, computer anxiety, and perceived enjoyment, all of which influenced the perceived utility and ease of using ICTs for learning. In Kubiatko and Vlckova’s (2010) study, students’ ICT use was favorably related to their scientific performance, especially when ICT was employed for instructional purposes.

In addition, Al-Rahmi et al. (2020a) discovered a substantial association between computer self-efficacy and subjective norms, which were important drivers of reported ease of use and perceived usefulness in influencing students’ intentions to use for education. According to Leino (2014), moderate and diverse ICT use can help the pupils improve their reading abilities, especially male students. In contrast to the findings of this research, which imply that ICT use in academic contexts has a favorable impact, because of the mixed results about the functions of ICT usage, it is critical to double-check prior findings using more rigorous models and statistical methodologies. Five factors (e.g., student interaction, student engagement, expected effort, subjective norm, and perceived ease of use) that positively affect both ICT usage in learning and academic success should be taken into account. The detrimental influence of ICT discovered in prior studies may be interpreted from a variety of angles.

For starters, there might be a disconnect between what is received through ICT and what is assessed (Pelgrum, 2001; Huang et al., 2021). In other words, it is possible that polls under-represent the potential benefits of using ICT in education. Second, students’ lack of basic ICT skills and abilities, as well as their desire to use ICT in the classroom, may be the contributing factors (Rohatgi et al., 2016; Sayaf et al., 2021).

The impact of ICTs on students’ interaction and engagement was shown to be the most closely associated factor with the ICTs’ use in active learning, according to the findings of this study. As a result, it was critical to investigate ways to improve students’ ICT abilities, capacities, engagement, and interaction through the use of ICTs for learning throughout the course design and execution. Furthermore, the effect of ICTs on the anticipated effort, subjective norm, and TAM model variables (perceived ease of use, perceived enjoyment, and perceived usefulness) was found to be the most closely connected factor with ICTs’ usage in active learning. It is likely that as computers and the Internet have grown more widely available, the opportunity gap between internet access and ICT use has narrowed, decreasing the link between active learning and ICT use in the classroom (Chiao and Chiu, 2018; Al-Rahmi et al., 2020a).

On the one hand, circumstances other than owning a computer at home are more likely to have a role in ICT use in learning. As previously noted, ICT self-efficacy and learning incentives may be more essential than having home access to computers and the Internet (Rohatgi et al., 2016; Sayaf et al., 2021). On the other hand, as new educational policies have been implemented across the Middle East, including Saudi Arabia, ICT has become more frequently employed in educational settings. ICT-based learning is now accessible even for pupils who do not have access to a computer at home.

This supports (Sayaf et al., 2021, 2022) results that ICT availability at schools is favorably connected to student academic progress, but ICT availability at home is adversely related. In other words, for ICT to be used to deliver explicit learning instructions, it must be viewed as both easy to use and beneficial. Furthermore, this study’s conclusions include that expected effort, subjective norm, and perceived ease of use affected perceived usefulness and perceived enjoyment, both belief constructs functioning as predictors for students’ active learning (Figure 4 and Table 6). As a result, this study demonstrates that students may use ICTs to improve their academic performance. Furthermore, our study has resulted in the development of a validated instrument to assess students’ active learning and real usage of ICTs in higher education. Finally, the scientific contributions are as follows:

• Regarding the independent factors hypothesis on the AUIs for learning in higher education, students’ interactivity, students’ engagement, expected effort, subjective norm, and perceived ease of use ICTs were found to affect perceived enjoyment and perceived usefulness of ICTs.
• Regarding the mediator factors hypothesis on the AUIs for learning in higher education, perceived enjoyment and perceived usefulness of ICTs were found to affect active learning, students’ attitude toward use, and behavioral intention to use ICTs for learning.
• Regarding the mediator factors hypothesis on the AUIs for learning in higher education, students’ active learning through ICTs was found to affect students’ attitude toward use and behavioral intention to use actual ICTs for learning.
• Regarding the dependent factors hypothesis on the AUIs for learning in higher education, students’ attitude toward use and behavioral intention to use ICTs were found to affect the AUIs for learning.

### Conclusion and Future Work

The purpose of this study was to empirically investigate the development of a validated instrument to measure students’ active learning and AUIs for learning in Saudi Arabia’s higher education. As a result, the purpose of this study was to look into the impact of ICTs on learning outcomes, as well as to anticipate and look into the factors that impacted students’ behavioral intentions to use ICTs for learning, as well as their AUIs for learning.

The findings demonstrated that utilizing ICTs may give students with positive learning benefits and that students’ interaction, engagement, expected effort, subjective norm, and perceived ease of use can all affect perceived utility and enjoyment. Furthermore, active learning had significant mediating effects between perceived usefulness, perceived enjoyment, students’ attitude toward use, and behavioral intention to use ICTs for education; in other words, perceived usefulness primarily influenced perceived enjoyment, whereas active learning influenced students’ attitude toward use, behavioral intention to use, and AUIs for education. While the recent research has significant ramifications, it is not without flaws.

It should be noted that we have only looked at 10 essential elements that influence students’ active learning, attitudes toward usage, and behavioral intention to use using an SEM analytical technique. Previous contradictory findings that have sparked much controversy about the impact of ICTs on students’ academic success could be linked to the complex, dynamic surroundings and other demographic characteristics, so future research should look at the possible contributions of both individual and environmental factors. Previous contradictory findings that have sparked much controversy about the impact of ICTs on students’ academic success could be linked to the complex, dynamic surroundings and other demographic characteristics (Hu et al., 2018; Huang et al., 2021).

## Data Availability Statement

The original contributions presented in the study are included in the article/supplementary material, further inquiries can be directed to the corresponding author.

## Ethics Statement

Ethical review and approval was not required for the study on human participants in accordance with the local legislation and institutional requirements. Written informed consent from the patients/participants or patients/participants legal guardian/next of kin was not required to participate in this study in accordance with the national legislation and the institutional requirements.

## Author Contributions

The author confirms being the sole contributor of this work and has approved it for publication.

## Conflict of Interest

The author declares that the research was conducted in the absence of any commercial or financial relationships that could be construed as a potential conflict of interest.

## Publisher’s Note

All claims expressed in this article are solely those of the authors and do not necessarily represent those of their affiliated organizations, or those of the publisher, the editors and the reviewers. Any product that may be evaluated in this article, or claim that may be made by its manufacturer, is not guaranteed or endorsed by the publisher.
